# Early detection in oral cancer: are we ready for AI-driven precision?

**DOI:** 10.1097/MS9.0000000000004418

**Published:** 2025-12-03

**Authors:** Sinha Kumari, Nikil Kumar, Muhamma Saad Khan, Sadia Sultana, Muddassir Khalid

**Affiliations:** a Department of Medicine, Jinnah Sindh Medical University, Karachi, Pakistan; b Department of Medicine, Liaquat University of Medical and Health Sciences, Jamshoro, Pakistan; c Department of Medicine, Azad Jammu and Kashmir Medical College, Muzafarabad, Pakistan; d Department of Medicine, Nishtar Medical University, Multan, Pakistan

**Keywords:** artificial intelligence, oral cancer care, personalized treatment

## Abstract

Oral cancer, particularly oral squamous cell carcinoma, remains a serious health concern, with a poor prognosis and a late diagnosis. Leukoplakia, erythroplakia, lichen planus, and submucous fibrosis are examples of oral potentially malignant illnesses. However, traditional diagnostic approaches are typically laborious, subjective, and unreliable, leading to delayed diagnosis – when therapy options are limited and survival is compromised.

In oral cancer, artificial intelligence (AI) and precision medicine are becoming game-changing technologies that enhance individualized care, treatment planning, and diagnostic precision. Machine learning and deep learning algorithms, particularly convolutional neural networks, can analyze massive, complex datasets from fluorescence to hyperspectral imaging, revealing patterns that are beyond human detection.

Recent trials have shown AI systems based on smartphones have demonstrated expert-level accuracy in identifying oral lesions in recent experiments. Through the discovery of biomarkers and the integration of several omics, AI-driven precision medicine also makes customized treatments possible.

Nonetheless, issues with patient privacy, data bias, and the opaque “black box” nature of AI systems persist. The future of proactive and individualized oral cancer therapy relies on creating Explainable AI and strong ethical frameworks that encourage transparency, trust, and equitable integration.

*Dear Editor,*

Oral cancer remains one of the most challenging malignancies, with poor prognosis and substantial morbidity despite advances in therapy^[1]^. The most common form, oral squamous cell carcinoma (OSCC), is often preceded by oral potentially malignant disorders, such as leukoplakia, erythroplakia, lichen planus, and submucous fibrosis^[2]^. Detecting these disorders early is critical, yet conventional diagnostic tools such as oral examination, biopsy, and histopathology are slow, subjective, and prone to fluctuation. The ongoing reliance on these procedures contributes to late diagnosis, when therapeutic options are restricted and survival is compromised. This manuscript follows the TITAN guidelines to explore the use of artificial intelligence (AI) and precision medicine for the diagnostic, therapeutic, and early detection of OSCC^[3]^.

AI and precision medicine represent a revolutionary prospect in oral oncology. AI-powered technologies are improving diagnostics’ precision, repeatability, and treatment planning^[4]^. Machine learning and deep learning algorithms can analyze enormous, complicated datasets – from fluorescence to hyperspectral imaging – revealing patterns that are beyond human detection^[2]^. Convolutional neural networks, in particular, hold promise for automating histopathology and integrating multimodal imaging, but their reliance on large, homogeneous datasets raises questions regarding bias and generalizability^[5]^.

Notably, a recent trial assessing a deep learning system installed on a smartphone for the detection of oral cancer and possibly malignant conditions demonstrates the usefulness of AI. The system was evaluated in three different settings: independently, aiding dentists, and supporting noncertified health care, and attained diagnostic accuracy comparable to an expert^[6]^. In actuality, these innovations allow for more accurate tumor delineation, earlier discovery, and treatment plans customized to the molecular and genetic characteristics of each patient^[4]^.

Potentialities extend beyond diagnosis. Precision medicine, aided by AI, is poised to transform oral cancer therapy. Algorithm-driven analytics and predictive biomarkers can direct immunotherapy and tailored treatments, boosting effectiveness and lowering toxicity. While biomarker-driven precision medicine uses single molecular fingerprints to guide targeted therapeutics, systems biology recognizes the complexity of interconnected networks across genomes, proteomics, and clinical characteristics. AI has the unique opportunity to improve both by expediting biomarker development and integrating multi-omics to illuminate the larger tumor ecology via deep learning. Having the ability to match patients with the most effective strategy based on their individual tumor biology has the potential to transform oral oncology from a reactive field to a proactive one^[4]^.

Although there is a lot of promise for AI in oncology, the “black box” nature of many AI systems remains a significant ethical concern. It is crucial to protect patient data privacy, reduce algorithmic bias by using a variety of datasets, improve model explainability, and build up explicit legal frameworks. The creation of Explainable AI (XAI) is essential to guaranteeing transparency and confidence in AI-driven decision-making^[7]^. XAI is very important for making critical judgments, especially in clinical settings when making the wrong choice could have catastrophic effects, including the loss of human life^[8]^.

The future of AI in oral oncology will be marked not just by smarter algorithms but also by their seamless integration into clinical care, equal access across populations, and open, accountable use^[2]^. AI and precision medicine, when combined with doctors, scientists, and policymakers, have the potential to transform oral cancer from a disease of late discovery and bad outcomes to one of earlier detection, better treatment, and more equitable management^[9]^. The tools are here – the urgency now resides in our collective determination to act. TITAN Guidelines: This manuscript is in compliant to the TITAN Guidelines, 2025, declaring no use of AI^[3]^.

## Data Availability

Not applicable.
